# Initiation of Low-Dose Intravenous Buprenorphine for Opioid Use Disorder: A Case Series and Literature Review

**DOI:** 10.7759/cureus.68007

**Published:** 2024-08-28

**Authors:** Praveen Reddy Elmati, Hira Waseem, Gowthami Sai Kogilathota Jagirdhar, Christhopher M Stewart, Alexander Bautista

**Affiliations:** 1 Anesthesiology, Saint Clare’s Health, Denville, USA; 2 Psychiatry, University of Louisville School of Medicine, Louisville, USA; 3 Internal Medicine, Saint Michael's Medical Center, Newark, USA; 4 Anesthesiology, University of Louisville Hospital, Louisville, USA

**Keywords:** opioid management, pain management, opioid use disorder, buprenorphine, addiction medicine

## Abstract

Opioid use disorder (OUD) remains a significant public health challenge with patients often facing barriers to initiating medications for opioid use disorder (MOUD). Traditional initiation methods for buprenorphine-naloxone (buprenorphine/naloxone) can be challenging due to the longer duration of transition and the risk of precipitated withdrawal. This manuscript presents a case series of four patients who successfully transitioned to buprenorphine/naloxone maintenance using a novel approach: low-dose intravenous (IV) buprenorphine initiation. These cases presented in the manuscript involved patients with dual diagnoses of OUD and difficult-to-treat pain. Intravenous buprenorphine was administered at a dose of 0.3 mg every half-hour, with a maximum of four doses. Patients' withdrawal symptoms were monitored using the Clinical Opioid Withdrawal Scale (COWS). Comfort medications were provided as needed. All four patients were successfully transitioned to sublingual (SL) buprenorphine/naloxone without experiencing precipitated withdrawal. Patients were discharged with follow-up appointments at buprenorphine/naloxone clinics and bridge supplies of buprenorphine/naloxone. Low-dose IV buprenorphine initiation offers a rapid and effective method for transitioning patients from full-agonist opioids (FAOs) to buprenorphine/naloxone without precipitated withdrawal. This approach has the potential to increase treatment retention and patient satisfaction. This case series highlights the success of low-dose IV buprenorphine initiation in patients with OUD and chronic pain. Further research is needed to standardize this approach and assess long-term outcomes. Initiating MOUD with this method may improve patient care and reduce the burden on the healthcare system.

## Introduction

Buprenorphine/naloxone is one of the first-line treatment options for opioid use disorder (OUD), but its initiation process can be quite challenging [1]. Transitioning from full-agonist opioids (FAO) to maintenance buprenorphine/naloxone carries the risk of precipitated withdrawal. This can be a strong deterrent for most patients. Various society guidelines recommend tapering and discontinuing FAO for at least 36-72 hours before initiating buprenorphine/naloxone [1, 2]. This waiting period is intended to allow the clearance of these FAO from the body before starting buprenorphine/naloxone treatment. Initiating buprenorphine/naloxone therapy too soon after discontinuing FAO can precipitate withdrawal symptoms due to the partial agonist properties of buprenorphine and its high affinity for opioid receptors [3]. Precipitated withdrawal occurs when buprenorphine displaces FAO from the receptors, leading to a sudden decrease in opioid activity, which can result in severe withdrawal symptoms.

In contrast, nonpharmaceutical fentanyl, especially short-acting formulations, has a relatively shorter duration of action compared to long-acting opioids. As such, the waiting period before initiating buprenorphine/naloxone therapy for individuals using nonpharmaceutical fentanyl may be shorter compared to long-acting opioids, but it still depends on individual factors such as the dosage, frequency, and duration of fentanyl use [3]. 

Holding FAO in patients with acute pain and opioid withdrawal can be challenging due to several factors. Patients with acute pain may require continuous FAO therapy for adequate pain relief. Discontinuing FAO abruptly in these patients can lead to uncontrolled pain, which poses a clinical challenge. Abrupt cessation of FAO can lead to the onset of withdrawal symptoms, which can be distressing for patients and may require additional management strategies to address withdrawal symptoms. The onset and severity of withdrawal symptoms can vary widely among individuals based on factors such as opioid tolerance, duration of opioid use, and underlying medical conditions. Patients may experience anxiety, restlessness, irritability, gastrointestinal distress, tachycardia, and diaphoresis. Managing these variations effectively requires careful monitoring and individualized treatment approaches. The reasons above may cause fear or reluctance to undergo a period of opioid withdrawal, which can impact their willingness to engage in treatment. Engaging patients in shared decision-making and providing support and education about the process can help overcome some of these barriers [1, 2]. 

Low-dose buprenorphine using the sublingual (SL) formulation is an alternative approach used mostly in the outpatient setting [1]. Using intravenous (IV) buprenorphine as an alternative to SL buprenorphine allows for more rapid initiation, which can be especially valuable in the hospital setting. Buprenorphine is administered intravenously at the lowest dose compared to SL buprenorphine due to its higher bioavailability when given intravenously. By administering IV buprenorphine at 0.3 mg dosage, clinicians can achieve a similar initial plasma concentration while avoiding potential complications associated with rapid increases in drug levels [4-7]. This approach is particularly valuable in the hospital setting, where rapid initiation of buprenorphine/naloxone therapy may be necessary for managing acute opioid withdrawal and/or providing analgesia.

To address the possibility of precipitated withdrawal, the concept of using low-dose, also called “micro-dosed” intravenous buprenorphine to facilitate a rapid transition to SL buprenorphine-naloxone, bypassing precipitated withdrawal while still effectively engaging opioid receptors, is a valuable treatment option. It may also be an easier and shorter process, requiring less utilization of healthcare resources. 

Buprenorphine has a high affinity for opioid receptors but acts as a partial agonist, meaning it produces less activation of opioid receptors compared to FAOs like heroin or oxycodone. By administering low doses of buprenorphine, gradual receptor occupancy occurs, allowing for a smoother transition and minimizing the abrupt displacement of pre-existing FAO from opioid receptors, which can trigger precipitated withdrawal. Buprenorphine exhibits a ceiling effect on respiratory depression and euphoria, which means that at higher doses, these effects plateau even with increasing doses. Micro-dosing allows for careful titration of buprenorphine dosage based on individual patient response. Despite all these concerns for withdrawal, very large doses have been studied in the ED in OUD patients without issues [8]. The objective of this case series is to present four cases that were successfully transitioned to buprenorphine/naloxone maintenance with the use of IV buprenorphine.

## Case presentation

The selection of all mentioned cases was based on a collaborative approach involving the addiction psychiatry service and the pain medicine department. Patients chosen for inclusion had the dual diagnosis of chronic pain and opioid use disorder diagnosed by the Diagnostic and Statistical Manual of Mental Disorders, Fifth Edition (DSM-5) criteria and using substance abuse and mental health services administration guidelines [3]. Methadone was considered for the treatment of patients but was not used due to prior failure or strict compliance around establishing and maintaining care at a methadone clinic, which was difficult for some patients. The dose of IV buprenorphine administered to patients was determined primarily by pain physicians, relying on their clinical expertise and prior literature review [4-7]. The dose used was 0.3 mg at half-hour intervals, with an average of three to four doses of IV buprenorphine administered. Intravenous buprenorphine 0.3 mg is the approved dose for acute pain management (can be repeated at 30-60 minutes) and is bioequivalent to about 1 mg sublingually. The decision to administer IV buprenorphine at half-hour intervals takes into consideration the pharmacokinetics of buprenorphine. When administered intravenously, buprenorphine typically reaches peak plasma concentration within five to 15 minutes. This rapid onset of action allows for relatively quick attainment of therapeutic levels in the bloodstream. In contrast, SL administration of buprenorphine results in a slower onset of action, with peak plasma concentration typically occurring around one to two hours after administration. By spacing the doses at half-hour intervals, healthcare providers can closely monitor the patient's response and titrate the dosage accordingly to achieve the desired therapeutic effect without precipitating withdrawal or other adverse effects. Furthermore, the decision to administer an average of three to four doses of IV buprenorphine is based on clinical judgment, considering Case Two. 

Factors such as the severity of opioid dependence and the need to balance the rapid initiation of buprenorphine therapy with the risk of adverse effects or precipitated withdrawal. Witnessed informed written consent was taken from the patients for conducting the study and publishing it. These case series are presented as aggregate data and without patient identifiers.

Case one

A 21-year-old male with a medical history of type 1 diabetes mellitus (T1DM), OUD, intravenous drug use (IVDU), multiple unintentional overdoses, and repeated admissions for diabetic ketoacidosis (DKA) was admitted again with DKA. The patient had a remote history of being on methadone but with poor adherence and intermittent periods of return to use. The patient’s most recent return to use was three to four months before the admission. The last heroin and fentanyl use was within 24 hours of hospital admission. The addiction psychiatry team was consulted to offer buprenorphine/naloxone initiation, but the patient declined it due to the fear of precipitated withdrawal. Finally, the pain management team was consulted to attempt to help overcome the barrier of precipitated withdrawal through IV buprenorphine initiation. The patient was subsequently started on IV buprenorphine, 0.3 mg, half hourly for three doses. The following day, the patient was initiated on SL buprenorphine/naloxone. No precipitated withdrawal was noted either during IV buprenorphine initiation or sublingual buprenorphine/naloxone initiation. The patient was started on 2 mg of buprenorphine/naloxone, and the dose was titrated up every six to eight hours gradually to a final dose of 24 mg over three to four days. The patient was monitored for withdrawal symptoms through the Clinical Opiate Withdrawal Scale (COWS) protocol throughout the process. The COWS score ranged between four and nine. Comfort medications for withdrawal symptoms, including gabapentin 300 mg every eight hours (q8), one clonidine patch 0.1 mg, hydroxyzine 25 mg q6 hourly as needed, Zofran 4 mg q6 hourly as needed, and diazepam 5 mg/IV one-time dose for anxiety, were started. The patient was discharged on buprenorphine/naloxone 24/6 mg every morning (qAM), mirtazapine 15 mg nightly, and gabapentin 300 mg q8 hourly. The social work team assisted with setting up outpatient follow-ups at a buprenorphine/naloxone clinic. A bridge prescription of buprenorphine/naloxone was provided.

Case two

A 39-year-old male with OUD and IVDU was admitted for increasing lower back and bilateral lower extremity intractable pain with an L4-S1 posterior compressive epidural abscess. The patient underwent L4-5 laminectomy and evacuation of epidural abscess. The patient had recently returned to using fentanyl before admission. Before return-to-use, he was taking prescribed buprenorphine/naloxone 12 mg daily for three months. The patient expressed interest in transitioning back to buprenorphine/naloxone but feared precipitated withdrawal and poor pain control with buprenorphine/naloxone; thus, he declined the plan. He consistently reported poor pain control despite being on hydromorphone patient-controlled analgesia (PCA) since the procedure. He ultimately agreed with the plan to transition to buprenorphine/naloxone. Still, the idea of potentially holding opioid pain medications before standard buprenorphine/naloxone initiation seemed very daunting to the patient. We decided to use microdose IV buprenorphine initiation to minimize the risk of precipitated withdrawal. Buprenorphine was initiated intravenously at 0.3 mg (total of three doses). Comfort medications as needed (PRN) for opioid withdrawal symptoms and pain, including oral oxycodone 5 mg q4 hourly, 0.5 mg IV hydromorphone q8 hourly, gabapentin 600 mg q8 hourly, and IV toradol 30 mg q6 hourly PRN, were provided. The COWS protocol was used to monitor for opiate withdrawal, and the COWS score ranged around eight. Patients tolerated the IV buprenorphine very well without precipitated withdrawal. The patient was started on sublingual buprenorphine/naloxone the day following IV buprenorphine initiation and discharged on a total daily dose of buprenorphine/naloxone of 12 mg (8 mg qAM and 4 mg every evening (qPM)). The social work team assisted with setting up an appointment at the buprenorphine/naloxone clinic, and a bridge prescription for buprenorphine/naloxone was provided.

Case three

A 58-year-old male with a medical history of T1DM, unspecified cardiac disease, opiate use disorder, and hepatitis C virus (HCV) presented as a transfer case from an outside hospital due to concerns for bowel ischemia and acute liver failure. He was found to have septic shock and DKA. Additionally, the patient had undergone traumatic intubation and developed dysphagia, hoarseness, and loss of vocal cord function bilaterally. A percutaneous endoscopic gastrostomy (PEG) tube was placed initially, later replaced with a gastrostomy tube (G tube). The patient had severe pain throughout the admission and was maintained on IV hydromorphone and oxycodone for pain. Psychiatry was consulted to initiate buprenorphine/naloxone initiation, but the patient declined initially due to fear of poor pain control with buprenorphine/naloxone. When close to discharge, the primary team consulted the psychiatry team again, given that the patient continued to receive high doses of IV hydromorphone with limited benefit for pain, and this was also a potential barrier for him to be accepted to a physical rehabilitation facility. The patient finally agreed to initiate buprenorphine/naloxone but was unwilling to try sublingually given that SL initiation required him to be off all opiates for at least eight to 10 hours. He was given the option of IV buprenorphine initiation, to which he agreed, and the chronic pain/anesthesia team was consulted to assist with IV buprenorphine initiation. The patient was given PRN medications for opiate withdrawal, including a clonidine patch, which caused an episode of hypotension. Clonidine was held. The patient received 3X doses of 0.3 mg IV buprenorphine every 30 minutes. Three additional PRN doses of 0.3 mg IV buprenorphine were given four and nine hours after the initiation for ongoing pain. The patient was then initiated on SL buprenorphine/naloxone 8 mg twice a day (BID) the following morning. No precipitated withdrawal symptoms were reported either during IV or SL initiation. Additional doses of buprenorphine are given only for ongoing pain. The buprenorphine/naloxone dose was titrated fairly quickly given the severity of the reported pain. Buprenorphine/naloxone titration was done as follows: 8 mg BID on day one, then 12 mg BID followed by 8 mg thrice a day (TID) for better pain coverage. The patient was discharged to an inpatient physical rehab facility.

Case four

The fourth case is of a 34-year-old female with a history of OUD, amphetamine use disorder, and HCV who was admitted with 10%-15% total body surface area (TBSA) burns to the abdomen and thighs and underwent multiple debridement. The patient was reportedly intoxicated on heroin and fell asleep next to an open flame burner and awoke with clothes on fire. The patient required high doses of FAOs throughout admission. The patient's pain regimen included Tylenol, oxycodone PRN, gabapentin, robaxin/methocarbamol, hydroxyzine PRN, and PRN IV hydromorphone for dressing changes. Addiction psychiatry was consulted during the initial days of admission, and the plan regarding buprenorphine/naloxone initiation had to be delayed until the patient would no longer need debridement. The patient had tried buprenorphine/naloxone in the past with a brief period of sobriety and was interested in trying it again. The patient continued to require dressing changes twice a day, which can be quite painful. The chronic pain team was consulted to manage the patient's pain and attempted IV buprenorphine initiation. The patient received a total of two doses of IV buprenorphine 0.3 mg that were spaced by about eight hours, one clonidine patch 0.1 mg, and oral hydroxyzine 25 mg twice in 24 hours. She denied any precipitated withdrawal throughout the process. She was transitioned to receiving SL buprenorphine/naloxone 8 mg TID the following day. Buprenorphine/naloxone doses were timed 30 minutes before each dressing change (for burns). She reported good pain control and did not require additional opioid pain medications. She was discharged to a residential treatment facility that offers both substance use treatment and medical treatment.

Using the IV formulation of buprenorphine micro-dosing, we could successfully initiate patients. Patients were monitored on the COWS protocol. Comfort medications for withdrawal, including clonidine, ondansetron, gabapentin, and hydroxyzine, were provided. All patients underwent a successful transition to SL buprenorphine-naloxone the following day without experiencing precipitated withdrawal. The patients reported improvements in cravings, withdrawal symptoms, and some relief from pain. Additionally, all four patients were scheduled for outpatient medications for opioid use disorder (MOUD) appointments and provided with a bridge supply of buprenorphine/naloxone.

## Discussion

The current standard of care for opioid use disorder involves medications such as buprenorphine, methadone, and naltrexone, collectively known as MOUD [1]. However, numerous barriers hinder the initiation of MOUD, including limited access to healthcare providers who can prescribe these medications. In the case of buprenorphine-naloxone initiation, the risk of precipitated withdrawal poses additional obstacles to successful treatment. Previous studies have already established that low-dose initiation of SL buprenorphine is associated with fewer adverse effects and better treatment retention compared to standard initiation methods. Traditional low-dose buprenorphine/naloxone usually takes up to two weeks, while in our method we could successfully transition in two days. Few case reports in previous literature report this technique [6, 9]. One strength of this approach is the speed of initiation. This approach offers several advantages, including a reduced abstinence requirement, rapid initiation, and the absence of precipitated withdrawal. By enabling patients to transition quickly to SL buprenorphine/naloxone, they have a higher likelihood of achieving sobriety and a reduced risk of unintentional overdose upon leaving the hospital.

While the benefits of micro-dosed SL buprenorphine initiation have been well-established in previous studies [10], there is limited evidence regarding the advantages of using intravascular buprenorphine for rapid initiation within a shorter period. The exact physiological mechanisms of IV buprenorphine micro-initiation are not yet fully understood. It may be due to a potential reduction in the proportion of displaced mu receptors or differences in bioavailability [11].

In the presented case series, all four patients had a long history of opioid use disorder and secondary medical conditions associated with chronic pain. When severe opioid use disorder is complicated by pain, the initiation of bupropion/naloxone becomes even more challenging. A significant number of patients unknowingly use fentanyl, which has a longer clearance time from the body due to its high lipophilicity. This poses greater challenges for buprenorphine initiation, leading to lower retention rates and higher return-to-use rates. Patients often struggle to tolerate the required period of abstinence before safely administering buprenorphine, resulting in poor compliance and a perception of treatment ineffectiveness. The presented case series demonstrates the successful transition of all four patients to SL buprenorphine/naloxone using low-dose IV buprenorphine initiation during their inpatient hospital admission. Notably, the initiation process occurred without the precipitation of opioid withdrawal symptoms and did not necessitate patients discontinuing opioid pain medications for any significant period. An important outcome of this case series is the achievement of more successful buprenorphine initiation, increased patient satisfaction, and the establishment of MOUD before discharge.

Hardy M et al. conducted a survey on low-dose buprenorphine initiations, which was utilized by 25 healthcare institutions [12]. The authors state that only 4% of the institutions used IV buprenorphine initiation and transition to SL buprenorphine. The dose of IV buprenorphine commonly used by institutions was 300 mcg q6 hourly on day one, followed by SL buprenorphine/naloxone 2 mg q6 hourly on day two, and 4 mg q6-8 hourly on day three.

We searched the literature on PubMed and the Excerpta Medica database (EMBASE) for low-dose IV buprenorphine initiation for OUD using keywords such as ("buprenorphine") AND ("intravenous") AND ("microdose*" OR "low-dose" OR "low dose") AND ("opioid use disorder"). We found only two studies [13, 14], one case series [6], and two case reports [15, 9] of patients using the IV route. Table 1 shows the current literature using IV buprenorphine for initiation.

**Table 1:** Studies and case reports on low-dose IV buprenorphine initiation for OUD IV: intravenous; IQR: interquartile range; OUD: opioid use disorder; MME: morphine milligram equivalents; q6h: every six hours; UDS: urine drug screen; q2h: every two hours; q4h: every four hours; SL: sublingual; PRN: as needed

Serial No.	First Author	Country	Year of publication	Study design	Number of patients	Indications for low-dose buprenorphine	IV buprenorphine dosage used	Buprenorphine SL transition dose	Duration from initiation to transition median (IQR)	Median MME dose prior to initiation median (IQR)	Successfully transitioned to buprenorphine N (%)
Studies											
1	Murray et al. [13]	USA	2023	Retrospective case series	33	OUD requiring full agonist opioids for pain control	Standard regimen 27 patients: buprenorphine IV 0.15 mg q6h on day 1 with dose titration to 0.3 mg and then 0.4 mg on subsequent days.	SL buprenorphine-naloxone 2/0.5 mg twice daily on day 3 or 4	96 (72-96)	30.8 (15–60)	30 (90.9%)
							Slow regimen 6 patients: buprenorphine IV 0.15 mg dosed q12 h				
2	Jablomski et al. [14]	USA	2022	Retrospective study	59	(1) OUD and acute pain requiring full-agonist opioids, (2) OUD with recent non-prescribed fentanyl exposure as determined by UDS or patient report, and (3) OUD with recent methadone exposure.	Rapid dosing: buprenorphine IV 0.3 mg q6h x 2 doses followed by buprenorphine IV 0.6 mg q6h x 2 doses	Buprenorphine 4 mg single dose	41 (32–48)	120 (71–390 )	54 (91.5%)
							Moderate dosing: buprenorphine IV 0.15 mg q6h x 2 doses followed by Buprenorphine IV 0.3 mg q6h x 2 doses and buprenorphine IV 0.6 mg q6h x 2 doses.				
							Slow dosing: buprenorphine IV 0.15 mg q6h x 4 doses followed by buprenorphine IV 0.3 mg q6h x 2 doses and buprenorphine IV 0.6 mg q6h x 2 doses				
Case reports											
1	Illga et al. [15]	USA	2022	Case report	1	OUD	NA	SL buprenorphine-naloxone 8/2 mg twice daily	96	NA	1 (100%)
2	Crane et al. [9]	USA	2021	Case report	1	OUD	Buprenorphine IV 0.3 mg q6h	SL buprenorphine q2 hourly	96	NA	1 (100%)
3	Thakrar et al. [6]	USA	2022	Case reports	2	OUD	Buprenorphine IV 0.15 mg q6h with dose titration to 0.3 mg and then 0.4 mg on subsequent days	SL buprenorphine 4-8 mg q4-6h PRN	96	NA	2 (100%)

The scope of our case series was restricted to patients within a medical inpatient setting who were evaluated by the addiction psychiatry and pain medicine consult and liaison services. The successful IV initiation of buprenorphine required collaborative efforts between the pain medicine and addiction psychiatry teams. However, the limitation of our case series is that we did not conduct follow-up assessments with patients after their discharge to determine if they maintained remission from OUD.

Medication for OUD has the potential to prevent numerous deaths caused by overdose and other medical complications related to opioid use disorder. Patients with OUD often experience difficulties associated with IVDU, such as unintentional overdose, pneumonia, sepsis, septic joints, osteomyelitis, endocarditis, thromboembolism, pulmonary embolism, stroke, cardiac events, and chronic pain. This case series provides hope that microdosed IV buprenorphine initiation could be a novel approach for initiating MOUD (buprenorphine/naloxone) in patients with opioid use disorder. Additionally, this method allows for rapid initiation, which can help alleviate the burden on the healthcare system. Further studies should include follow-up to determine if this induction method is more effective than the traditional method and to standardize protocols. 

## Conclusions

This case series highlights the success of low-dose IV buprenorphine initiation in patients with OUD and chronic pain. Intravenous buprenorphine micro-dosing is an innovative method that can be used in hospitals to initiate rapid buprenorphine/naloxone initiation while minimizing and bypassing the risk of precipitated withdrawal. Further research on appropriate dosing and outcomes during initiation and in the long term is necessary in these patients.
